# Functional Characterization of Tissue Inhibitor of Metalloproteinase-1 (TIMP-1) N- and C-Terminal Domains during *Xenopus laevis* Development

**DOI:** 10.1155/2014/467907

**Published:** 2014-01-30

**Authors:** M. A. Nieuwesteeg, J. A. Willson, M. Cepeda, M. A. Fox, S. Damjanovski

**Affiliations:** Department of Biology, University of Western Ontario, 1151 Richmond Street, London, ON, Canada N6A 5B7

## Abstract

Extracellular matrix (ECM) remodeling is essential for facilitating developmental processes. ECM remodeling, accomplished by matrix metalloproteinases (MMPs), is regulated by endogenous tissue inhibitors of metalloproteinases (TIMPs). While the TIMP N-terminal domain is involved in inhibition of MMP activity, the C-terminal domain exhibits cell-signaling activity, which is TIMP and cell type dependent. We have previously examined the distinct roles of the *Xenopus laevis* TIMP-2 and -3 C-terminal domains during development and here examined the unique roles of TIMP-1 N- and C-terminal domains in early *X. laevis* embryos. mRNA microinjection was used to overexpress full-length TIMP-1 or its individual N- or C-terminal domains in embryos. Full-length and C-terminal TIMP-1 resulted in increased lethality compared to N-terminal TIMP-1. Overexpression of C-terminal TIMP-1 resulted in significant decreases in mRNA levels of proteolytic genes including TIMP-2, RECK, MMP-2, and MMP-9, corresponding to decreases in MMP-2 and -9 protein levels, as well as decreased MMP-2 and MMP-9 activities. These trends were not observed with the N-terminus. Our research suggests that the individual domains of TIMP-1 are capable of playing distinct roles in regulating the ECM proteolytic network during development and that the unique functions of these domains are moderated in the endogenous full-length TIMP-1 molecule.

## 1. Introduction

Remodeling of the extracellular matrix (ECM) is a dynamic process that is necessary for normal development [1]. Matrix metalloproteinases (MMPs) are a large family of 24 Zn^2+^-dependent endopeptidases that cleave various components of the ECM leading to changes in cell signaling and cell movement that are required during development [2]. Controlled inhibition of MMP activity is needed to prevent excessive ECM degradation and is largely carried out by the tissue inhibitors of metalloproteinases (TIMPs), as well as the cell surface MMP inhibitor REversion-inducing Cysteine-rich protein with Kazal motifs (RECK) [3]. Maintaining proper balance between MMP activity and inhibition is important during embryogenesis and facilitates many developmental events including organogenesis and angiogenesis [4, 5], and disrupting this balance can have deleterious effects [6, 7]. Inhibition of MMP-2 or -9, two potent ECM proteases, can lead to axial defects and incomplete neural crest cell migration, respectively [7, 8]. Knockout of membrane-bound MMP-14 (MT1-MMP) during mouse development is embryonic lethal [8–10], as is knockout of RECK due to defects in angiogenesis [11]. Additionally, our lab has shown that overexpression of TIMP-2 and -3 during *Xenopus laevis* development leads to axial and neural tube defects [12]. Thus, disruption of activity levels in this proteolytic ECM remodeling network is detrimental during development.

TIMPs constitute a family of only 4 secreted proteins (TIMP 1–4), which bind MMPs in a 1 : 1 manner to inhibit their proteolytic activity [13]. The four mammalian TIMPs share similar domain structure and 40% amino acid sequence similarity, including 12 conserved cysteine residues which result in the formation of 6 disulfide bonds [14]. TIMPs consist of structurally and functionally distinct N- and C-terminal domains, each of which is stabilized by 3 disulfide bonds [14]. The N- and C-terminal domains boundary is found between cysteine 6 and 7, in between which there are only one or two amino acids. The N-terminal domains of all TIMPs function to inhibit MMP activity by binding to the zinc-active site found in the catalytic domain of all MMPs [15]. The N-terminal domain of a TIMP is both necessary and sufficient for MMP inhibition [15]. In contrast, the smaller C-terminal domain of TIMPs may influence cell behavior in an MMP-independent manner through direct regulation of a number of cell-signaling pathways [16]. Characterization of cell surface binding partners and specific signaling events involving TIMP C-terminal domains has been carried out *in vitro*, and activation of a resultant signaling pathway seems to vary with the specific TIMP expressed as well as the cell type it is expressed in [17].

Of the four mammalian TIMPs, TIMP-1, -2, and -4 all function pericellularly, while TIMP-3 can be sequestered away from the cell surface in the ECM [18, 19]. TIMP-1 was first identified for its erythroid potentiating activity and as an inhibitor of metalloproteinases [20, 21]. Both the N- and C-terminal domains of TIMP-1 have now been well characterized *in vitro*. Interestingly, the two domains of TIMP-1, as well as of the other TIMPs, have the ability to fold and function autonomously *in vitro* [15, 22]. TIMP-1 can inhibit MMP-2 and -9 activity, which are two powerful ECM proteases that cleave abundant ECM components [3, 23]. Additionally, TIMP-1, through its C-terminal domain, has been linked to regulation of specific cell-signaling pathways. TIMP-1 has been shown to promote cell proliferation, although the receptor-mediated events involved in this pathway remain unidentified [24]. More recently, TIMP-1 has been associated with inhibition of apoptosis in several human cell lines [25, 26]. TIMP-1 may also increase the abundance of cellular survival and differentiation factors, and this activity has been linked to association of TIMP-1 with cell surface CD63 and *β*1 integrin [25, 27–29]. To date, however, the specific activities of the individual TIMP-1 domains have not been well characterized *in vivo*, and the specific roles of the two domains as they pertain to development remain unknown.

Our lab has previously preformed a series of domain specific overexpression experiments, which suggest that TIMP-2 and -3 N- and C-terminal domains may have specific MMP-dependent and independent functions, respectively, during embryogenesis in *X. laevis *[12]. In the present study, we use a similar approach to characterize the unique functions of the TIMP-1 N- and C-terminal domains during early *X. laevis* development. Here we show that the TIMP-1 C-terminal domain can act autonomously to alter gene expression and MMP levels in *X. laevis* embryos, and that axial and head defects resulting from C-terminal domain overexpression are different than those observed with the N-terminal domain.

## 2. Materials and Methods

### 2.1. Cloning of *X. laevis* TIMP-1

*X. laevis* TIMP-1 was cloned using primers designed from unannotated *X. laevis* clone AAI41767.1. Primers used were forward: 5′ACAGAAGGACTGCCCAGCC and reverse: 5′CAAAACACTTCTCCTTCGAG. *Xenopus *TIMP-1 was cloned from total cDNA from stage 35 embryos using SuperScript Reverse Transcriptase (Life Technologies) with Platinum *Taq* DNA Polymerase High Fidelity (Life Technologies). The full-length TIMP-1 amplicon was cloned into the pCR II-TOPO vector (Life Technologies), and the sequence of full-length *X. laevis* TIMP-1 was confirmed at the Robarts Research Institute DNA Sequencing Facility, at the University of Western Ontario, and submitted to Genbank as KF018236.

### 2.2. Comparison of Amino Acid Identity between Vertebrate TIMP-1 N- and C-Terminal Domains

Sequence alignments and amino acid identities were compared using all annotated full-length TIMP-1 sequences known at the time of publication. Accession numbers were as follows: human (*Homo sapiens*) NP_003245.1; mouse (*Mus musculus*) AAH51260.1; rat (*Rattus norvegicus*) EDL97726.1; cow (*Bos Taurus*) AAP44413.1; horse (*Equus Caballus*) NP_001075984.1; rabbit (*Oryctolagus cuniculus*) AAW79053.1; shark (*Callorhinchus milii*) AFK11387.1; newt (*Notophthalmus viridescens*) ABB88702.1.

### 2.3. Animals and Ethics Statement

Adult *X. laevis* were purchased from Ward's Natural Science (Rochester, NY). Fertilization and rearing of embryos were done in accordance with standard protocols [30] and staged according to Nieuwkoop and Faber [31]. Animals were housed and treated in accordance with UWO and CCAC guidelines. The protocol was approved by University of Western Ontario Animal Use Subcommittee/University Counsel on Animal Care, AUP number 2009-44.

### 2.4. Generation of TIMP-1 mRNA Constructs for Microinjection

PCR was used to generate full-length, N-terminal, or C-terminal TIMP-1 (hemagglutinin) HA-tagged constructs using the full-length clone as a template. Briefly, all TIMP-1 mRNA constructs (T1FL, T1N, and T1C) were HA tagged on their most C-terminal end. The C-terminal domain construct (T1C) was engineered to include its appropriate secretory signal sequence through a two-step PCR process. Forward and reverse primers used were as follows:

T1FL 5′AGATCTATGTTGTACCTTGTGGTTGTG;

5′CAGTCTGCTGCCACAACACAATACCCATACGATGTTCCAGATTACGCTACTAGT;

T1N 5′AGATCTATGTTGTACCTTGTGGTTGTG;

5′GTGTATCGCAAAGCCTGTTCCTACCCATACGATGTTCCAGATTACGCTACTAGT;

T1C signal sequence 5′AGATCTATGTTGTACCTTGTGGTTGTG;

T1C signal sequence link 5′CTCAGCCAGGAGGTGTTGGGGTGCAACATCGTCCCCTGCTAT;

T1C-terminal domain link 5′TGCAACATCGTCCCCTGCTAT;

5′CAGTCTGCTGCCACAACACAATACCCATACGATGTTCCAGATTACGCTACTAGT.

Following PCR amplification, the sequences of all amplicons were verified. All TIMP-1 constructs were subsequently ligated into the *EcoRV/SpeI* restriction sites of the T7TS plasmid. mMessage mMachine was used with T7 RNA polymerase (Ambion) to synthesize stable capped, poly(A)-tailed mRNA transcripts that were dissolved in filtered water. RNA was quantified using NanoVue spectrophotometer (GE), and its integrity was assessed using 1% agarose gel electrophoresis. GFP mRNA was generated and quantified in a similar way from a proven T7TS-GFP construct plasmid [32]. The T7TS plasmid and HAs tag have previously been used extensively by our lab and others and their utilities have been demonstrated.

### 2.5. mRNA Microinjection

Prior to injection, *X. laevis *embryos at the one-cell stage were transferred from 0.1X (Marc's Modified Ringer's solution) MMR into 1X MMR containing 4% Ficoll. Embryos were injected at the one-cell stage with TIMP-1 full-length, N-terminal, or C-terminal (T1FL, T1N, and T1C) constructs, or GFP mRNA, using 10 *μ*m diameter glass needles. Embryos were injected with 4 ng of mRNA in a volume of 2.3 nL. Embryos were maintained in 1X MMR containing 4% Ficoll for 5 hours following injection and then transferred to 0.1 X MMR solution for rearing. Embryos that were dead or abnormal 2 hours after injection were removed, and the remaining embryos (representing the 100% being monitored) were observed for phenotypic abnormalities. The percent of normal embryos (no visible defects) was quantified for two days following injection (until stage 30 in development). Mean values were obtained from three independent sets of experiments.

### 2.6. Semiquantitative RT-PCR Analysis

Semiquantitative RT-PCR analysis was used to examine changes in proteolytic gene expression patterns or to confirm upregulation of TIMP-1 expression following injection with T1FL, T1N, or T1C constructs compared to control embryos. Total RNA was extracted from 10 injected and control embryos using RNeasy Mini Kit (Qiagen) and 1 *μ*g RNA was reversely transcribed using qScript cDNA Supermix (Quanta Bioscienes). PCR was carried out using KAPA Taq PCR Kit (KAPA Biosystems) and the Eppendorf Thermal Cycler. The cycling conditions were as follows: initial denaturation, 95°C for 2 min, followed by 28 cycles of the following: denature 95°C 30 sec, anneal 55°C 30 sec, and extend 72°C 30 sec. Primers, based on the *X. laevis* sequences, were as follows:

Ef1*α*  5′TGTTGGCAGAGTGGAGACTG and 5′GGCCAAGTGGAGGATAGTCA;

TIMP-2 5′AGGTAAAGCCGATGGTGATG and 5′GCCTGTCGTCCGTTTATGTT;

TIMP-3 5′TTCCAAGAACGAGTGCCTCT and 5′GGGATCCGTGGTGTTTATTG;

RECK 5′GGATGTTTACAGGTCTACCC and 5′GGCTCTGTTCCTCCAAAGAT;

MT1-MMP 5′CTATGAGGCGATTCGGAGAG and 5′GCCACCAGGAACAGATCATT; MMP-2 5′TGATTCTGGTCGCTCAGATG and 5′CTTGTTTCCCAGGAAGGTGA;

MMP-9 5′AGGACCATGGGGATCCTTAC and 5′AACACAAGGCTGCCCATTAC;

T1FL and T1C 5′ATGTTGTACCTTGTGGTTGT and 5′TTATTGTGCTGTGCAGCAG;

T1N 5′ATGTTGTACCTTGTGGTTGT and 5′GGAACAGGCTTTGCGATACAC.

### 2.7. Protein Preparations and Western Blotting

Stage 30 embryos injected with T1FL, T1N, or T1C mRNA constructs, or uninjected controls, were subject to protein extraction. Briefly, 10 embryos were lysed and sonicated in 100 *μ*L modified RIPA buffer (150 mM NaCl, 50 mM tris (pH 8.0), 1.0% NP-40, 0.5% sodium deoxycholate, 0.1% SDS, 10 *μ*L/mL Halt Protease Inhibitor Cocktail (Thermo Scientific), and 10 *μ*L/mL Halt Phosphatase Inhibitor Cocktail (Thermo Scientific)) and lysates were centrifuged at 15,000 g for 25 min at 4°C. The supernatant was removed and protein was quantified using BCA protein assay kit (Thermo Scientific) according to manufacturer's instructions. 25 *μ*g of protein was electrophoresed on a 10% SDS gel and transferred to PVDF membrane (Bio-Rad). Membranes were washed three times with TBST (Tris-buffered saline containing 0.5% Tween-20), blocked in 5% skim milk in TBST for 40 min at room temperature, and incubated overnight at 4°C with primary rabbit anti-HA antibody (1 : 1000 dilution; Santa Cruz) or mouse anti-*β*-actin antibody (1 : 1000 dilution; Santa Cruz). Membranes were washed 3 times in TBST and incubated with secondary antibodies goat anti-rabbit HRP (1 : 5000 dilution; Life Technologies) or goat anti-mouse HRP (1 : 5000 dilution; Bio-Rad), respectively, for 1 hour at room temperature. Membranes were washed 3 times in TBST and HRP activity was detected and visualized using SuperSignal West Pico Chemiluminescent Substrate (Thermo Scientific) and Bio-Rad Quantity One 4.4.0 software.

### 2.8. Zymography and Reverse Zymography

12.5 *μ*g of protein (as extracted for Western blotting) from T1FL, T1N, or T1C injected embryos, or uninjected control embryos, was diluted 1 : 1 with 2X SDS-loading buffer (0.5 M Tris-HCl, pH 6.8, 10% SDS, 2.5% glycerol, and 1% Bromophenol Blue). For zymography, protein samples were electrophoresed on a 1% gelatin and 10% polyacrylamide gel. For reverse zymography, protein was electrophoresed on a 1% gelatin and 15% polyacrylamide gel copolymerized with MMP-conditioned media from Hs578t cells according to [33]. Hs578t cells were chosen for their ability to secrete high levels of MMPs, particularly MMP-2 and -9. For both zymography and reverse zymography, gels were electrophoresed in 1X zymogram running buffer (25 mM Tris, 192 mM glycine, and 0.1% SDS), followed by in-gel renaturation for 30 min at room temperature in renaturing solution (2.5%Triton X-100). Substrate cleavage was carried out by incubating gels in zymogram developing buffer (pH 7.5, 50 mM Tris, 200 mM NaCl, 5 mM CaCl_2_ (anhydrous), and 0.02% Brij-35) for 48 hours at 37°C. For zymography, MMP activity was visualized as clear bands against a dark background after staining with 0.5% Coomassie blue (Bio-Rad). For reverse zymography, TIMP activity was visualized as dark bands against light background after staining with 0.5% Coomassie blue (Bio-Rad). Gels were visualized and photographed using Bio-Rad Quantity One 4.4.0 software.

### 2.9. Statistical Analysis

All statistical analysis was performed using the IBM SPSS Statistic 19 program. Results were presented as mean ± standard error. Statistical significance was determined using one-way ANOVA variance analysis and Dunnett's post hoc test. Differences were considered statistically significant when *P* < 0.05.

## 3. Results

### 3.1. Amino Acid Sequence Conservation of Vertebrate TIMP-1 N- and C-Terminal Domains

**Figure 1:**
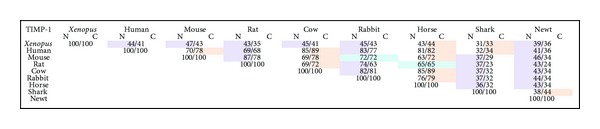
Evolutionary conservation of TIMP-1 N- and C-terminal domains. Sequence analysis comparing amino acid sequence identity of *X. laevis* TIMP-1 N- and C-terminal domains versus known (at the time of publication) vertebrate species. Light purple boxes represent species whose N-terminal domains were more highly conserved with *X. laevis* TIMP-1 than their C-terminal domains. Light pink boxes represent species whose C-terminal domains were more highly conserved with *X. laevis* TIMP-1 than their N-terminal domains. Blue boxes represent species with equal conservation with both *X. laevis* TIMP-1 N- and C-terminal domains.

A direct amino acid sequence comparison of the individual TIMP-1 N- and C-terminal domains between species has not previously been reported. Current TIMP-1 protein sequences were identified, which thus far have only been confirmed in vertebrates. To investigate whether the unique functions of these domains may be concurrent with their patterns of evolutionary conservation, we cloned *X. laevis* TIMP-1 (GenBank: KF018236) and compared its N- and C-terminal domains with currently known mammalian and nonmammalian TIMP-1 sequences. The N-terminal domain of *X. laevis* TIMP-1 was more highly conserved than its C-terminal domain with 7 of 9 vertebrate species analyzed (Figure 1). In general, the N-terminal domains of all species were more highly conserved than the C-terminal domains, with the exception of horse and cow, where the C-terminal domains were more highly conserved between species (Figure 1).

### 3.2. TIMP-1 RNA and Protein Were Upregulated in *X. laevis* Embryos following Ectopic Expression of TIMP-1 mRNA Constructs

**Figure 2:**
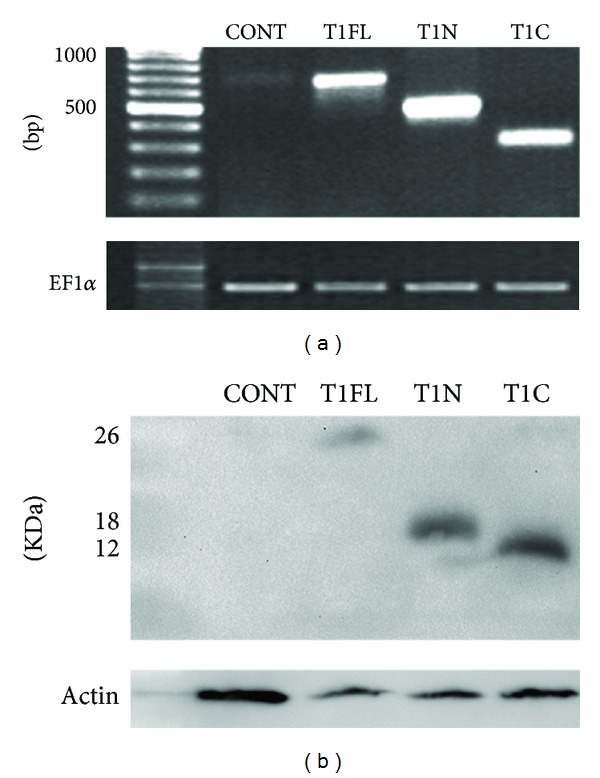
Confirmation of full-length, N-terminal, or C-terminal TIMP-1 constructs overexpressed in *X. laevis *embryos. 4 ng of mRNA coding for full-length (T1FL), N-terminal (T1N), or C-terminal (T1C) TIMP-1 constructs was microinjected into *X. laevis *embryos at the 1-cell stage. (a) Overexpression of TIMP-1 mRNA as shown by RT-PCR analysis. RNA was isolated from stage 15 embryos. Control (uninjected) embryos expressed TIMP-1 at very low levels. Primers specific to each construct were used to confirm mRNA levels of T1FL (678 bp), T1N (453 bp), and T1C (252 bp). RT-PCR of EF1*α* was used as a loading control. (b) Increased levels of TIMP-1 constructs as shown by Western blot analysis. All constructs are HA tagged. Protein was isolated from stage 30 embryos. Anti-HA antibodies were used to confirm expression of each construct at the protein level (T1FL = 26 KDa, T1N = 18 KDa, and T1C = 12 KDa). No HA was detected in control uninjected embryos. *β*-actin was used as protein loading control.

In order to characterize the unique roles of TIMP-1 N- and C-terminal domains during development, we have performed a series of domain specific overexpression experiments. For this purpose, we generated full-length, N-terminal, and C-terminal HA-tagged TIMP-1 mRNA constructs (T1FL, T1N, and T1C, resp.), which were injected into newly fertilized* X. laevis *embryos (1-cell stage). Total RNA was collected from injected and control embryos 1 day after fertilization (stage 15), and RT-PCR analysis was used to verify levels of TIMP-1 mRNA transcripts in injected embryos compared to controls (Figure 2(a)). Uninjected control embryos contained much lower levels of TIMP-1 mRNA compared to T1FL, T1N, or T1C injected embryos, which showed relatively equal but elevated levels of TIMP-1 mRNA, relative to Ef1*α* (loading control; Figure 2(a)). By two days after fertilization (stage 30), each HA-tagged TIMP-1 construct was still present and detectable on Western blot via HA antibody in injected embryos (Figure 2(b)), indicating that all constructs produced stable protein products.

### 3.3. Overexpression of Full-Length, N-Terminal, and C-Terminal TIMP-1 Constructs Produced Unique Developmental Phenotypes

**Figure 3:**
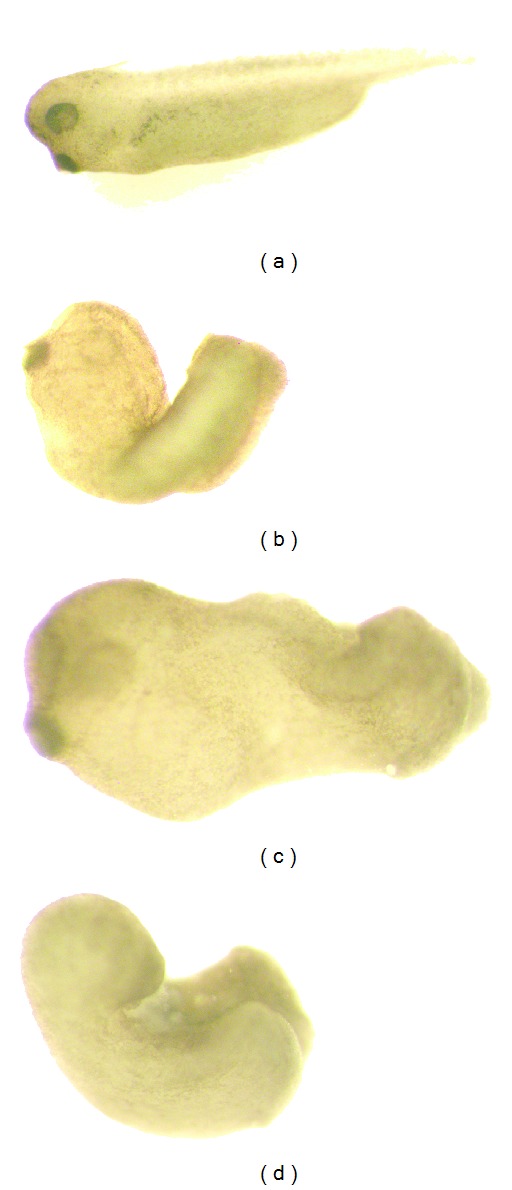
Phenotypic effects of overexpression of full-length, N-terminal, or C-terminal TIMP-1 constructs. Following injection, photographs were taken of representative embryos at stage 30. (a) Control (uninjected) embryos were phenotypically normal. Microinjection of T1FL (b) and T1N (c) constructs both resulted in normal anterior (head) development but truncated posterior axes. (d) Microinjection of T1C constructs resulted in lack of head structures, other head defects, and neural tube closure failure. Panel (a) magnification is lower than in (b), (c), and (d). Embryos are 1 mm in diameter.

Following injection of each TIMP-1 construct, embryos were monitored for gross morphological changes in development for 2 days after fertilization (until developmental stage 30). Uninjected control embryos were morphologically normal (Figure 3(a)). By stage 30 in development, embryos overexpressing full-length (T1FL) or N-terminal (T1N) TIMP-1 displayed relatively normal anterior (head) development with posterior axis defects. T1FL and T1N injected embryos had a bent axis phenotype along with truncated anterior/posterior axes compared to controls (Figures 3(b) and 3(c), resp.). Embryos overexpressing C-terminal TIMP-1 (T1C) also resulted in truncated axis defects compared to controls, as well as other developmental abnormalities: chiefly, lack of head structures, other head defects, and failure of the neural tube to close (Figure 3(d)). More specifically, overexpression of T1C resulted in more severe phenotypic consequences than overexpression of T1FL or T1N constructs.

### 3.4. Overexpression of Full-Length and C-Terminal TIMP-1 Resulted in Increased Embryonic Lethality Compared to Overexpression of N-Terminal TIMP-1

**Figure 4:**
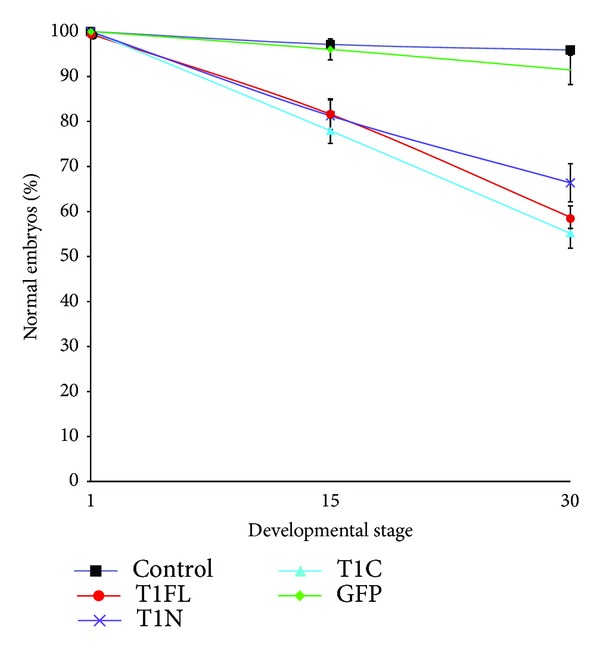
Overexpression of all three TIMP-1 constructs leads to abnormal development and death. Following injection of mRNA constructs at the 1-cell stage, embryos were scored for a normal phenotype at stages 15 and 30. Dead and abnormal embryos were counted as containing morphological defects. The graph illustrates the percentage of normal embryos following injection of GFP mRNA (GFP), or full-length (T1FL), N-terminal (T1N), or C-terminal (T1C) TIMP-1 mRNA constructs at the given stage. Control embryos are uninjected. Results are based on 3 independent sets of experiments; bars indicate standard error.

To compare and measure the effects of overexpressing full-length versus N-terminal or C-terminal TIMP-1 during *X. laevis* development, T1FL, T1N, and T1C injected embryos were monitored for 48 hours following fertilization, and the percent of normal embryos (embryos that were alive and morphologically normal) was determined and quantified at stage 15 (1 day after fertilization) and stage 30 (2 days after fertilization). More than 90% of control embryos, or embryos injected with GFP (green fluorescent protein) mRNA, developed normally during this time period (Figure 4). Overexpression of all TIMP-1 constructs resulted in increased lethality compared to controls. At stage 15, all constructs resulted in an approximate 20% decrease in normal embryo numbers compared to controls at the same stage (Figure 4). By stage 30, 66% of T1N injected embryos developed normally, whereas T1FL and T1C constructs were more detrimental with only 58% and 55% normal development, respectively (Figure 4).

### 3.5. Overexpression of Full-Length, N-Terminal, or C-Terminal TIMP-1 Constructs in *X. laevis *Embryos Altered Expression of Proteolytic Genes

In order to examine the roles of full-length, N-terminal, or C-terminal TIMP-1 in regulating ECM remodeling *in vivo*, we investigated the expression patterns of hallmark genes involved in regulating ECM proteolysis following microinjection with T1FL, T1N, or T1C constructs into *X. laevis* embryos. mRNA was collected from embryos at stage 30 in development, as we had observed specific and unique morphological defects at this time point with overexpression of each construct (see Figure 3). Using semiquantitative RT-PCR, we assayed for changes in mRNA levels of the MMP inhibitors (TIMP-2, TIMP-3, and RECK), as well as the MMPs (MMP-2, MMP-9, and MT1-MMP). All of these genes have previously been shown to be important regulators of ECM remodeling during development [8, 9, 12, 34]. mRNA levels were normalized to Ef1*α* and compared to control (uninjected) embryos.

**Figure 5:**
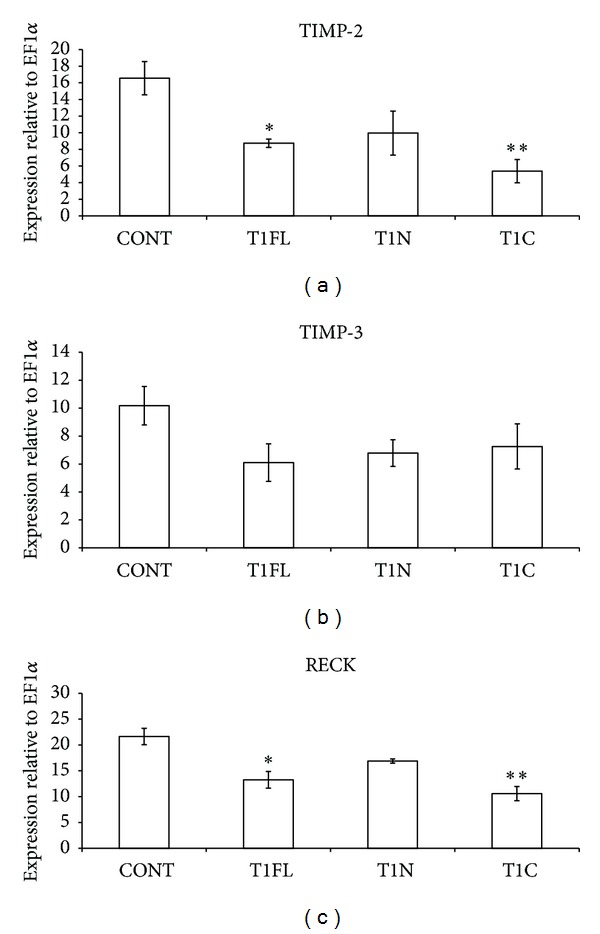
Effect of overexpression of full-length, N-terminal, or C-terminal TIMP-1 on mRNA levels of MMP inhibitors. Semiquantitative RT-PCR analysis was used to measure changes in mRNA levels of (a) TIMP-2, (b) TIMP-3, and (c) RECK at stage 30, following microinjection of 4 ng of TIMP-1 full-length (T1FL), N-terminal (T1N), or C-terminal (T1C) constructs into *X. laevis *embryos at the 1-cell stage. In each case mRNA levels were measured relative to Ef1*α*. The results are presented as mean ± standard error from 3 independent experiments. *P < 0.05 and **P < 0.01, all versus control (CONT; uninjected) embryos, as analyzed by one-way ANOVA and Dunnett's post hoc test.

Overexpression of T1C significantly decreased TIMP-2 mRNA relative to controls (Figure 5(a), *P* < 0.05). T1FL and T1N did not significantly alter TIMP-2 mRNA, although the trend was toward the decrease for T1FL injected embryos. Levels of TIMP-3 mRNA were not significantly altered compared to control embryos following treatment with any of our TIMP-1 constructs (Figure 5(b)). RECK expression was significantly decreased relative to controls following injection of T1FL (*P* < 0.05) and T1C (*P* < 0.01, Figure 5(c)).

**Figure 6:**
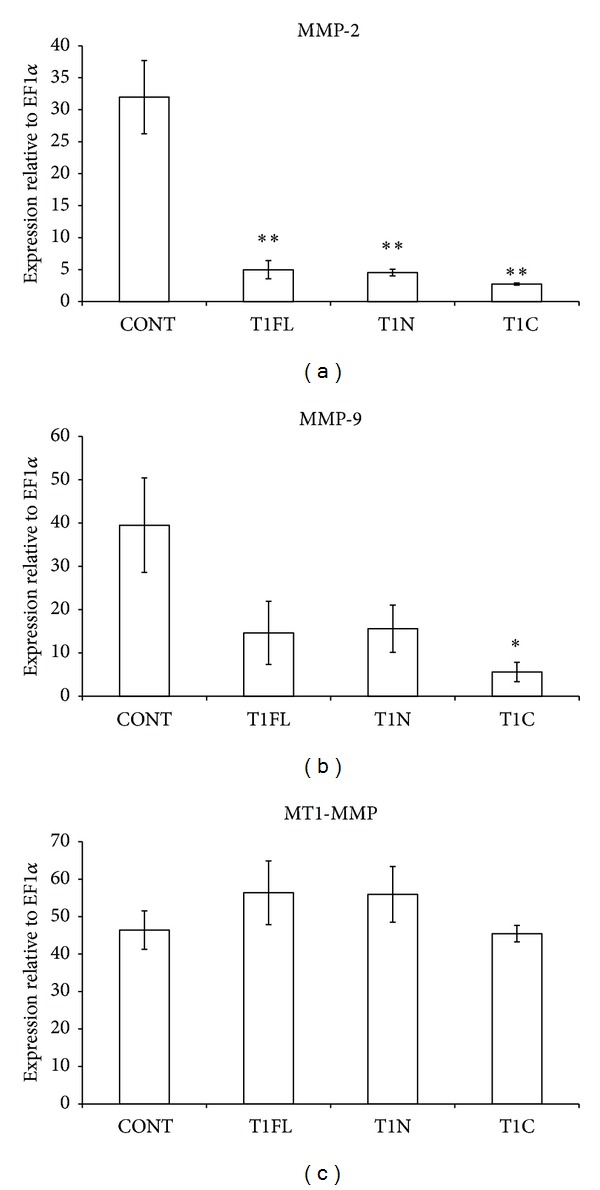
Effect of overexpression of full-length, N-terminal, or C-terminal TIMP-1 on mRNA levels of MMPs. Semiquantitative RT-PCR analysis was used to measure changes in mRNA levels of (a) MMP-2, (b) MMP-9, and (c) MT1-MMP at stage 30, following microinjection of 4 ng of TIMP-1 full-length (T1FL), N-terminal (T1N), or C-terminal (T1C) constructs into *X. laevis *embryos at the 1-cell stage. In each case mRNA levels were measured relative to Ef1*α*. The results are presented as mean ± standard error from 3 independent experiments. *P < 0.05 and **P < 0.01, all versus control (CONT; uninjected) embryos, as analyzed by one-way ANOVA and Dunnett's post hoc test.

MMP-2 mRNA levels were significantly decreased compared to controls following overexpression of all constructs (T1FL, T1N, and T1C; Figure 6(a), *P* < 0.01). MMP-9 mRNA levels significantly decreased following injection of T1C (Figure 6(b), *P* < 0.05). No significant change in MMP-9 mRNA levels was seen in embryos injected with T1FL or T1N constructs (Figure 6(b)). Similarly, levels of MT1-MMP mRNA were not altered relative to control embryos following overexpression of any of our TIMP-1 constructs (Figure 6(c)).

### 3.6. Overexpression of TIMP-1 Constructs Decreased Amount of Active MMP-2 and -9

**Figure 7:**
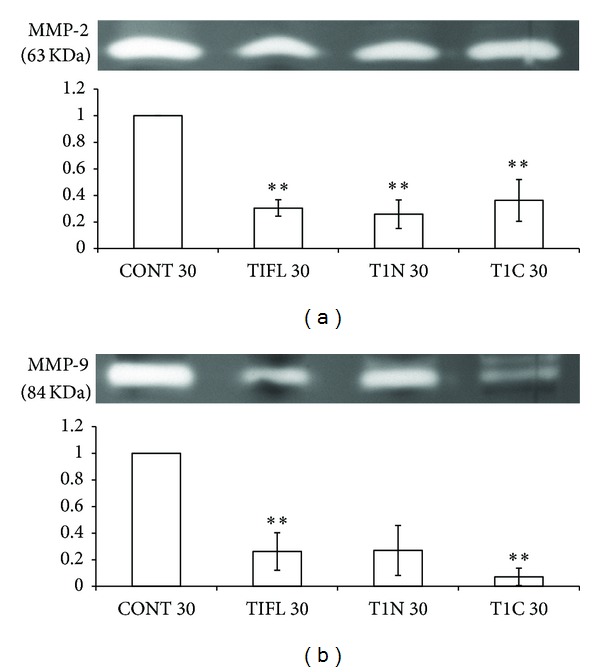
Zymography demonstrated altered levels of active MMP-2 and MMP-9 following overexpression of TIMP-1 constructs. 4 ng of mRNA coding for full-length (T1FL), N-terminal (T1N), or C-terminal (T1C) constructs was injected into *X. laevis *embryos at the 1-cell stage and protein was isolated from stage 30 embryos. Gelatin zymography was used to measure changes in (a) MMP-2 and (b) MMP-9 activity. Zymogram (top) is representative of one experiment where the bright brand represents the active forms (63 and 84 kDa) of MMP-2 and MMP-9, respectively. Graphs represent quantification of above zymogram, and data is presented as mean ± standard error from 3 independent experiments. *P < 0.05 and **P < 0.01, all versus control (CONT; uninjected) embryos, as analyzed by one-way ANOVA and Dunnett's post hoc test.

To further understand how the individual TIMP-1 domains affect the proteolytic network *in vivo*, gelatin zymography was used to measure changes in the amounts of active MMP-2 and -9 in T1FL, T1N, or T1C injected embryos relative to control (uninjected) embryos. Embryos injected with T1FL, T1N, and T1C constructs showed significantly decreased levels of active MMP-2 relative to control embryos (Figure 7(a), *P* < 0.01). Injection with T1FL and T1N constructs also resulted in decreased levels of embryonic active MMP-9 relative to controls (Figure 7(b), *P* < 0.05); however, interestingly, the T1C construct produced the most significant decrease in MMP-9 activity (Figure 7(b), *P* < 0.01).

### 3.7. Only Full-Length and N-Terminal TIMP-1 Domains Directly Inhibited MMP Activity

**Figure 8:**
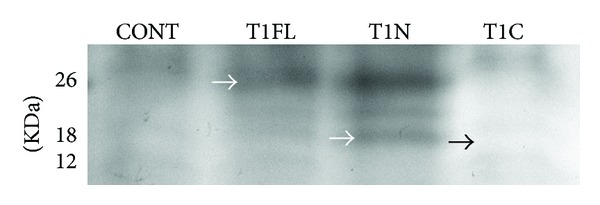
Reverse zymography demonstrating full-length and N-terminal, but not C-terminal, TIMP-1 can directly inhibit MMP activity. 4 ng of mRNA coding for full-length (T1FL), N-terminal (T1N), or C-terminal (T1C) TIMP-1 constructs was injected into *X. laevis *embryos at the 1-cell stage and protein was isolated from stage 30 embryos. MMP-inhibitory activity is represented by dark bands. White arrows indicate the location of the expected inhibitory band of the T1FL (26 KDa) and T1N (18 KDa) constructs, respectively. Black arrow represents position of T1C (12 KDa) construct, which cannot inhibit MMP activity as suggested by the light band.

In order to validate the MMP-inhibitory activity of our full-length and N-terminal TIMP-1 constructs and also to confirm that our C-terminal TIMP-1 construct cannot directly inhibit MMPs, we performed reverse zymography. Reverse zymography is a modification of traditional zymography by incorporating gelatin into the gel matrix as well as conditioned media from cancer cells as a source of MMP-2 and -9 activity. Subsequently, the proteins of interest are separated within the gel by molecular weight. The gelatin is degraded by MMPs in the matrix, except for where inhibited by active TIMPs. TIMP activity is visualized as a dark band after staining with Coomassie blue [33], representing a protected band corresponding to the molecular weight of the active construct. Protein was isolated from injected stage 30 embryos and analyzed for MMP-inhibitory activity corresponding to T1FL, T1N, and T1C construct sizes. T1FL and T1N constructs showed MMP-inhibitory activity at the expected sizes of 26 KDa and 18 KDa, respectively (Figure 8, white arrows), whereas the T1C construct was not able to inhibit MMPs at the expected size of 12 KDa (Figure 8, black arrow). Other bands represent the presence of other unknown inhibitors.

## 4. Discussion

ECM remodeling is a key process involved in regulating tissue growth and morphogenesis during development. Maintaining the appropriate balance between MMPs and their inhibitors (TIMPs and RECK) is absolutely essential for normal development, and disruption of this balance leads to defects in neurulation, organogenesis, and angiogenesis [4, 5, 8]. Our understanding of the regulation of this proteolytic network has become complicated by the discovery that TIMP proteins have both MMP-inhibitory activity and MMP-independent cell-signaling activity [35]. With recent research on TIMP C-terminal domains demonstrating their roles in apoptosis, cell proliferation, and cell migration pathways, the functions of the TIMP C-terminal domains are becoming increasingly studied *in vitro* [24–26, 36]. At the present time there is comparatively little research characterizing the roles of the N- and C-terminal TIMP domains *in vivo*, particularly as they pertain to development. Our lab has previously examined the specific functions of the N- and C-terminal domains of TIMP-2 and -3 during *X. laevis* development and found that the two domains have unique functions when overexpressed individually in *X. laevis* embryos [12]. To date, there has been no comparison of the unique functions of TIMP-1 N- and C-terminal domains in a developmental context. Here we use mRNA overexpression to examine the role of full-length TIMP-1 versus the roles of its individual N-terminal and C-terminal domains during early *X. laevis* development.

In order to examine whether distinct functions of the two TIMP-1 domains are reflected in their evolutionary conservation, we compared amino acid sequence identities of the *X. laevis* TIMP-1 N- and C-terminal domains to other known vertebrate TIMP-1 sequences. We found that the TIMP-1 N-terminal domains were more highly conserved than their C-terminal domains among most species analyzed, with the exception of the large mammals, horse, and cow, in which C-terminal domains were more highly conserved (Figure 1). As the catalytic domains of MMPs are highly conserved between species, it is not surprising that TIMP-1 N-terminal MMP-inhibitory domains are also well conserved [37]. We have previously shown that the TIMP-2 N-terminal domains are also more highly conserved across species than their C-terminal domains, whereas, for TIMP-3, we found that the C-terminal domains are more highly conserved [12]. Our findings suggest that TIMP-1 may behave more like TIMP-2 than TIMP-3 with respect to MMP inhibition. Indeed, TIMP-3 is unique in that it is a good inhibitor of the ADAM family of proteases [38]. Additionally, TIMP-3 binds to ECM components through its C-terminal domain and is sequestered away from the cell surface, whereas TIMP-1 and -2 function in a pericellular manner [39].

In order to examine differences in the *in vivo* roles of TIMP-1 N- and C-terminal domains, we generated and overexpressed full-length, N- and C-terminal TIMP-1 mRNA constructs in *X. laevis* embryos (T1FL, T1N, an T1C, resp.). We found that treatment of embryos with T1C resulted in more embryonic death and developmental defects than treatment with T1FL or T1N (Figure 4). Consistent with this observation, overexpression of T1C also resulted in more severe phenotypic defects in embryos by stage 30 in development, with these embryos showing head and neural tube defects, as well as truncated axes. In comparison, T1FL and T1N injected embryos showed relatively normal head development but truncated anterior-posterior axes (Figure 3), indicating that the TIMP-1 C-terminal domain may contribute to unique functions in development separate from the N-terminal domain.

In order to further investigate the roles of the two TIMP-1 domains, we performed RT-PCR analysis to examine changes in mRNA levels of associated genes involved in the regulation of ECM remodeling. We did not observe any change in levels of TIMP-3 or MT1-MMP mRNA relative to control embryos, following injection with full-length, N-terminal, or C-terminal TIMP-1. This is consistent with previous work, which showed that association of TIMP-1 with MT1-MMP is exceptionally poor [40]. In contrast, TIMP-2 and RECK mRNA levels decreased significantly compared to controls following treatment of embryos with T1FL (*P* < 0.05) or T1C (*P* < 0.01); however, these decreases were most pronounced with T1C (Figure 5). This supports the concept that the C-terminal domain has the ability to regulate developmental events independent of the N-terminal domain, presumably through cell surface receptor-mediated signaling pathways.

Similarly, we found that overexpression of T1C resulted in the most marked decreases in amounts of MMP-2 and -9 mRNA compared to control embryos, although, in the case of MMP-2, ectopic expression of all three constructs significantly decreased MMP-2 mRNA (Figure 6). These results were consistent with zymography, which showed that the amounts of active MMP-2 and -9 proteins followed the same trends as our RT-PCR analysis (Figure 7). Decreases in MMP-2 and -9 protein and mRNA may be partially due to a regulatory feedback mechanism in the proteolytic network which occurs from direct catalytic inhibition of MMPs by the N-terminal TIMP-1 domain, as T1N overexpression significantly decreased MMP-2 mRNA and protein compared to control embryos. Our results suggest, however, that the C-terminal domain of TIMP-1 has the ability to regulate MMP mRNA and protein levels independent of the N-terminal domain. Reverse zymography using MMP-conditioned media from Hs578t cells showed that TIC constructs cannot directly inhibit MMP activity (Figure 8); yet overexpression of T1C resulted in the most significant decreases in MMP-9 mRNA and protein compared to control embryos (Figures 6(b) and 7(b)). Although the mechanism of action of the TIMP-1 C-terminal domain is still unknown, TIMP-1 has previously been shown to bind to cell surface receptors on both fibroblasts and MCF-7 breast cancer cells, which resulted in translocation of TIMP-1 into the cell [40, 41]. Taken together with the emerging discoveries of new cell surface binding partners for TIMP-1 and its MMP-independent roles in cell signaling [42], it is possible that the C-terminal domain of TIMP-1 may function to regulate expression of proteolytic genes through a signaling mechanism separate from N-terminal MMP-inhibitory activity.

## 5. Conclusions

In summary, this research characterized *in vivo* the unique role of the TIMP-1 C-terminal domain. *In vitro* studies have demonstrated new roles for TIMP C-terminal domains in cell signaling. Additionally, disease models have highlighted the emerging significance of the C-terminal TIMP domains in regulating the proteolytic network, as synthetic MMP inhibitors designed to mimic TIMP N-terminal domains failed as an anticancer therapy to block metastasis associated with upregulated MMP activity [43]. Here we show for the first time *in vivo* that the TIMP-1 C-terminal domain has an independent role in regulating the proteolytic network during development, at a time when this network is both complex and highly regulated. We have no direct evidence as to the mechanisms through which the individual ectopic domains are manifesting developmental anomalies; however, there is evidence that feedback is involved as disruption of the precise embryonic proteolytic network was compensated for by changes in the levels of key genes. Further, while there is no evidence that nascent TIMP-1 is cleaved into its individual domains, our research suggests that the functions of the TIMP-1 N- and C-terminal domains depend on the affinities of the individual domains for their binding partners, as well as the stoichiometric levels of TIMP-1 relative to secreted MMPs and cell surface receptors. Further *in vivo* studies are needed to fully investigate whether there is preferential binding of either domain in the context of abundant active MMPs and/or cell surface receptors.
